# Computational study on novel natural inhibitors targeting c-MET

**DOI:** 10.1097/MD.0000000000027171

**Published:** 2021-09-24

**Authors:** Yuanyuan Hou, Haoqun Xie, Gaojing Dou, Wenzhuo Yang, Junliang Ge, Baolin Zhou, Junan Ren, Juncheng Li, Jing Wang, Zhiyun Zhang, Xinhui Wang

**Affiliations:** a Clinical College, Jilin University, Changchun, China; b Department of Breast Surgery, The First Bethune Hospital of Jilin University, Changchun, China; c Department of Gastroenterology, The First Affiliated Hospital of Xinxiang Medical College, Xinxiang, China; d Department of Oncology, First People's Hospital of Xinxiang, Xinxiang, China.

**Keywords:** c-MET, discovery studio, glioblastoma, tivantinib, virtual screening

## Abstract

This study was designed to select ideal lead compounds and preclinical drug candidates http://dict.youdao.com/w/eng/preclinical_drug_candidate/javascript:void (0); with inhibitory effect on c-MET from the drug library (ZINC database).

A battery of computer-aided virtual techniques was used to identify possible inhibitors of c-MET. A total of 17,931 ligands were screened from the ZINC15 database. LibDock is applied for structure-based screening followed by absorption, distribution, metabolic, and excretion, and toxicity prediction. Molecular docking was conducted to confirm the binding affinity mechanism between the ligand and c-MET. Molecular dynamics simulations were used to assess the stability of ligand-c-MET complexes.

Two new natural compounds ZINC000005879645 and ZINC000002528509 were found to bind to c-MET in the ZINC database, showing higher binding affinity. In addition, they were predicted to have lower rodent carcinogenicity, Ames mutagenicity, developmental toxicity potential, and high tolerance to cytochrome P4502D6. Molecular dynamics simulation shows that ZINC000005879645 and ZINC000002528509 have more favorable potential energies with c-MET, which could exist stably in the natural environment.

This study suggests that ZINC000005879645 and ZINC000002528509 are ideal latent inhibitors of c-MET targeting. As drug candidates, these 2 compounds have low cytotoxicity and hepatotoxicity as well as important implications for the design and improvement of c-MET target drugs.

## 1 Introduction

Glioblastoma (GBM) is the most common primary malignant brain tumor in the clinical central nervous system, which has a high degree of proliferation and invasion, accounting for 12% to 15% of all brain tumors.^[1]^ On average, 3.19 out of 100,000 people are diagnosed with malignant GBM every year, with an average age of 64 years.^[2]^ Currently, the standard treatment for patients with GBM is surgical resection and radiotherapy, accompanied by temozolomide or Carmustine chips. However, the heterogeneity and instability of GBM in its growth and differentiation make it prone to multiple resistance to radiation and chemical treatments.^[3]^ Therefore, the prognosis of most patients with GBM is poor and the therapeutic effect is not ideal.

c-MET is a member of the receptor tyrosine kinases superfamily, cell surface receptors that are heterodimers made up of a-chains and transmembrane d-chains (1145 k da) connected by disulfide bonds normally expressed in epithelial cells of various organs.^[4]^ c-MET is associated with a variety of oncogene products and regulatory proteins and has a strong role in promoting cell proliferation. It is involved in various processes in vivo, such as cell signal transduction and cytoskeletal reconstruction, and is an important factor in regulating the process of cell proliferation, differentiation, and repair. The c-MET gene encodes the receptor of the stem cell growth factor (HGF) ligand. In the classic HGF/c-MET signaling pathway, the binding of HGF to c-MET leads to the dimerization of c-MET and the self-phosphorylation of hydroxy-terminal tyrosine, thereby activating the downstream mitogen-activated protein kinase (MAPK) signaling pathway, phosphoinoside-3 kinase signaling pathway, and RAS-related C3 botox substrate 1-cell division cyclin 42 signaling pathway.^[5]^ c-MET is phosphorylated without HGF by binding to epidermal growth factor receptor, cell adhesion molecules, and abnormal prothrombin. Regardless of the pathway in which c-MET is activated, dimerization, phosphorylation, and kinase activation are necessary for malignant lesions in neurogenic tumors.^[6]^ c-MET was highly expressed in GBM cells, blood vessels, and peri-necrotic areas. At the subcellular level, c-MET was presented in the cytoplasm and in the cell membrane. c-MET has also been connected to the stem cell phenotype in glioma by regulating sphere formation, cell proliferation, and differentiation.^[7,8]^

In summary, c-MET kinase promotes GBM cell proliferation, migration, invasion, and angiogenesis. Therefore, the selection of effective c-MET kinase inhibitors plays an important role in drug development and cancer treatment. Currently, there are 2 kinds of c-MET inhibitors in the clinic. One is the monoclonal antibody against c-MET. One is small molecule kinase inhibitors. Tivantinib (ARQ197) is the first ATP competitive c-met inhibitor. Tivantinib selectively inhibits non-activated c-MET and inhibits self-phosphorylation of c-MET.^[9,10]^ Tivantinib inhibits c-MET by blocking the signaling cascade, promoting apoptosis, and inhibiting cell growth. However, there are some limitations. In some cases, Tivantinib failed 2 phase III studies involving second-line treatment of Met-high, advanced, hepatocellular carcinoma, despite its success during phase II studies.^[11]^ Consequently, the aim of this study was to screen natural compounds from natural drugs that are more effective than Tivantinib in treating cancer.

Natural products, as lead compounds, can be transformed into new drugs through appropriate structural modification, which is an important source of new drug research in the pharmaceutical industry.^[12,13]^ In recent years, several targeted drugs have been reported to inhibit c-MET.^[14]^ In this study, a series of structural biological and chemical methods (including virtual screening, molecular docking, etc) were used to screen and identify lead compounds with potential regulatory functions for c-MET. Our study also predicted the absorption, distribution, metabolism, excretion, and toxicity of these compounds. This study provides a list of drug candidates and their pharmacological properties, providing the research object for the development of c-MET inhibitors.

## 2 Method

### 2.1 Discovery studio software and ligand libraries

Discovery Studio is a suite of software designed to simulate small and large molecule systems, which is designed to screen, design, and modify potential drugs through structural chemistry and structural biology calculations, thereby identifying and refining a wide range of lead compounds and candidate drugs approaches. The LibDock and absorption, distribution, metabolism, and excretion (ADME) modules of Discovery Studio 4.5 software (DS4.5, Accelrys, Inc.) are applied in virtual screening. CDOCKER is used for docking research. Natural Products database in the ZINC database was used to screen c-MET inhibitors as a selection. The Irwin and Shoichet laboratories, which is in the department of pharmaceutical chemistry at the University of California, San Francisco, providing the ZINC database as a free commercial compound database.

### 2.2 Use LibDock for structure-based virtual filtering

**Figure 1:**
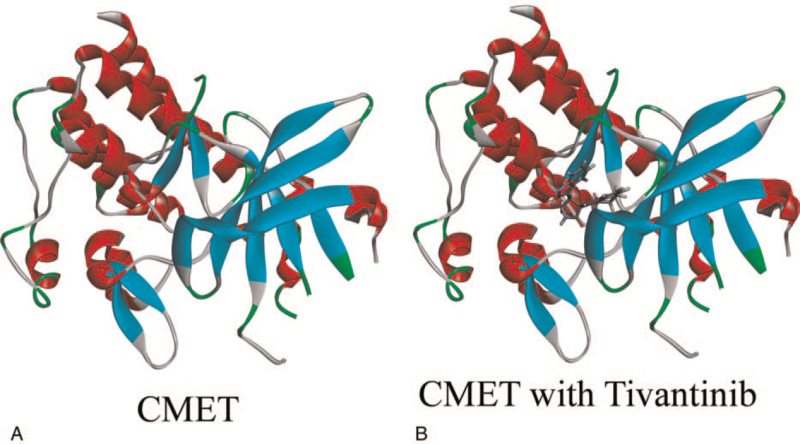
Molecular structure of c-Met. (A) Initial molecular structure. (B) Structure of binding area added. Blue represents positive charge and red represents negative charge.

The ligand-binding pocket region of c-MET was selected to identify new compounds that might inhibit c-MET as the binding site. Virtual filtering is performed using the LibDock module of Discovery Studio 4.5.^[15]^ LibDock is a rigid docking program. It uses grids placed at binding sites and polar and non-polar probes to calculate protein hotspots. To form favorable interactions, the hotspots are furtherly used to align ligands, as well as the Smart Minimiser algorithm and CHARMm force field (Cambridge, MA) for ligand minimization. All ligand positions were ranked by ligand scores after minimization. The 2.45 Å crystal structure of c-MET in conjunction with Tivantinib is downloaded from the protein database and imported into LibDock's work environment. The chemical structure of c-MET is shown in Figure 1. Proteins are made by removing crystalline water and other heteroatoms, and then adding hydrogen, protonation, ionization, and energy minimization. The CHARMm force field and Smart Minimiser algorithm were used to energy minimization.^[16]^ With a root mean square (RMS) gradient tolerance of 12.277, 2000 steps were performed in the minimization, which resulted in an RMS gradient of 0.09778. To define binding sites the prepared proteins were used, the Tivantinib binding site was selected as the active site for docking. Using LibDock, all prepared ligands were docked at defined active sites for virtual screening. According to the LibDock score, all docking positions are sorted and grouped by compound name.

### 2.3 ADME and toxicity prediction

The ADME module of Discovery Studio 4.5 is used to calculate the ADME of selected compounds, also used the DS4.5 toxicity prediction by Komputer assistive technology (TOPKAT) module to calculate all potential compounds toxicity and other properties, including its water-soluble, blood-brain barrier permeability, cytochrome P4502D6 (CYP2D6), liver toxicity, human intestinal absorption, plasma protein (PPB) levels, rodent carcinogenicity, Ames, respectively and developmental toxicity potential. These pharmacological properties should be taken into full consideration when selecting c-MET drug candidates.

### 2.4 Molecule docking and pharmacophore prediction

The CDOCKER module of Discovery Studio 4.5 is applied for molecular docking research. CDOCKER can produce high-precision docking results as a molecular docking method on the basis of CHARMm36 field. While allowing the ligand to bend during docking the receptor remains rigid. For each complex posture, the CHARMm energy (interaction energy plus ligand strain) and interaction energy indicate the ligand-binding affinity. From the protein database, we can obtain the crystal structure of c-MET. During rigid and semi-flexible docking processes, crystallized water molecules are generally removed for fixed water molecules may affect the formation of receptor-ligand complexes.^[17,18]^ Next, remove the water molecules and add the hydrogen atoms to the proteins. The initial compound, Tivantinib, was extracted from the binding site and then realigned into the crystalline structure of c-MET to demonstrate the reliability of the combination pattern. The force field of CHARMm36 is applied to the receptors and ligands. The definition of the binding site sphere of c-MET is that as the region within a radius of 16 Å from the geometric center of mass of the ligand Tivantinib. The ligand is combined with the residues in the binding spot during the docking. When it was ready to identify the hit structure, and docking it into the c-MET binding pocket, the CDOCKER process was performed.^[19,20]^ Based on CDOCKER interaction, different postures of each test molecule can be analyzed.

### 2.5 Molecular dynamics simulation

The best binding conformations of each compounds-c-MET complex were chosen for molecular dynamics simulation. an orthorhombic box was built for the ligand-receptor complex was put into an orthorhombic box and solvated with an explicit periodic boundary solvation water model. Solidum (ionic strength of 0.145) chloride was poured into the system for the sake of simulating the physiological environment. Then, the CHARMM force field and energy minimization were prepared for the system (500 steps of steepest descent and 500 steps of conjugated gradient), with result showing that the final RMS gradient of 0.227. The system was slowly driven from an initial temperature (296 K) to the target temperature (320 K) in 2 ps, and equilibration simulations were performed for 5 ps. Molecular dynamics simulation (production module) was run for 25 ps and the time step was 1 fs. The simulation was run with the normal pressure and temperature system (300 K) during the process. Long-range electrostatics were calculated by the particle mesh Ewald algorithm, and all bonds involving hydrogen were fixed by the linear constraint solver algorithm. Select initial complex setting as a reference, Discovery Studio 4.5 analysis trajectory protocol was used for a trajectory determined for RMS deviation (RMSD), potential energy, and structural characteristics.

Experiment to verify the therapeutic effect of the 2 selected compounds and c-MET expression in U251 cells.

### 2.6 Cell culture

U251 cell lines (GBM cell lines) were cultured in high glucose DMEM medium supplemented with 10% fetal bovine serum, cultured in a 37°C and 5% CO_2_ until the cells cover the bottom of the flask. Cells were passaged for just 1 time and cultured. The logarithmic growth phase of cells was selected for experimental use. Cell morphology was examined under a light microscope (Zeiss, Axiovert 200, Germany).

### 2.7 Detection of 2 selected compounds

U251 cells were seeded into 96-well plates at a density of 5 × 103/well, and each group had 3 duplicate wells. After 24 h, Tivantinib, Phyllanthin (ZINC5879645), and 4′-Hydroxycarvedilol (ZINC2528509) were added into 96-well plates with increasing drug concentrations and then cultured in 5% CO_2_ at 37°C for 72 h. The operation was carried out according to the instructions of the ELISA detection kit, and the c-MET expression of U251 cells was measured.

## 3 Result

### 3.1 Virtual screening of natural products database against c-MET

The ligand-binding pocket played an important part in the regulatory sites of c-MET. Therefore, we chose this pocket region as the reference site. A total of 17,931 ligands were screened from the ZINC15 database, which was marked as for-sale, biogenic, and named. Select the chemical structure of c-MET as the receptor to contrast the pharmacologic properties between it and other compounds. The compounds which scored in the top 20 were listed in Table 1.

**Table 1:** Top 20 ranked compounds with higher LibDock scores.

Number	Compounds	LibDock score
1	ZINC000004654840	149.287
2	ZINC000004654839	148.584
3	ZINC000049784088	145.293
4	ZINC000001531664	142.946
5	ZINC000014811789	141.38
6	ZINC000004098302	140.933
7	ZINC000011616633	139.868
8	ZINC000014614772	139.067
9	ZINC000002528509	138.818
10	ZINC000028882432	138.519
11	ZINC000013328774	137.446
12	ZINC000002526388	136.805
13	ZINC000006073947	136.732
14	ZINC000002528510	136.169
15	ZINC000013130933	135.767
16	ZINC000004098631	132.556
17	ZINC000004654839	131.474
18	ZINC000031163978	131.363
19	ZINC000005879645	130.843
20	ZINC000001763468	127.941

### 3.2 ADME and toxicity prediction

ADME module of Discovery Studio 4.5 was used to predict the Pharmacologic properties of the whole selected ligands with Tivantinib first, including aqueous solubility level, BBB level, CYP2D6 binding, human intestinal absorption level, hepatotoxicity, and PPB binding properties (Table 2). According to aqueous solubility prediction (defined in water at 25 °C), most of the compounds could dissolve in water. Three-quarters of the compounds were predicted to be noninhibitors CYP2D6, which had a great influence on drug metabolism. As for hepatoxicity, only 7 of 20 compounds were found to be nontoxic, the remaining compounds were poisonous which was similar to Tivantinib. For human intestinal absorption, 6 compounds and Tivantinib were predicted to have good absorption. PPB binding properties showed most compounds had weak absorption.

**Table 2:** Predicted adsorption, distribution, metabolism, and excretion properties of compounds.

Number	Compounds	Solubility level	BBB level	CYP2D6	Hepatotoxicity	Absorption level	PPB level
1	ZINC000004654840	0	4	0	0	3	1
2	ZINC000004654839	0	4	0	0	3	1
3	ZINC000049784088	4	4	0	0	3	0
4	ZINC000001531664	2	4	0	1	3	0
5	ZINC000014811789	3	4	0	1	3	0
6	ZINC000004098302	1	0	1	1	1	1
7	ZINC000011616633	2	4	0	0	3	0
8	ZINC000014614772	1	1	1	1	0	1
9	ZINC000002528509	2	4	1	1	0	1
10	ZINC000028882432	3	4	0	1	3	0
11	ZINC000013328774	3	4	0	1	3	0
12	ZINC000002526388	2	4	1	1	0	1
13	ZINC000006073947	2	2	0	0	0	1
14	ZINC000002528510	2	4	1	1	0	1
15	ZINC000013130933	1	4	0	1	2	1
16	ZINC000004098631	1	4	0	1	3	1
17	ZINC000004654839	0	4	0	0	3	1
18	ZINC000031163978	1	4	1	0	3	0
19	ZINC000005879645	2	1	0	1	0	1
20	ZINC000001763468	2	4	0	1	1	1
21	Tivantinib	1	2	1	1	0	0

BBB = blood–brain barrier, CYP2D6 = cytochrome P-450 2D6, PPB = plasma protein binding. Aqueous-solubility level: 0, extremely low; 1, very low, but possible; 2, low; 3, good. BBB level: 0, very high penetrant; 1, high; 2, medium; 3, low; 4, undefined. CYP2D6 level: 0, noninhibitor; 1, inhibitor. Hepatotoxicity: 0, nontoxic; 1, toxic. Human-intestinal absorption level: 0, good; 1, moderate; 2, poor; 3, very poor. PPB: 0, absorbent weak; 1, absorbent strong.

**Figure 2:**
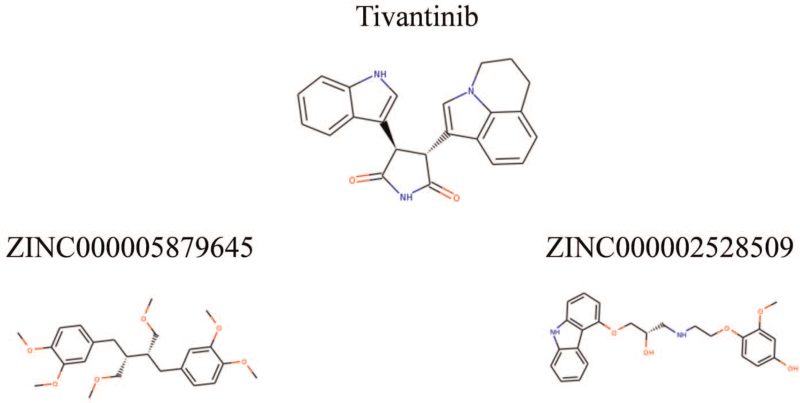
Structure of c-Met and novel compounds selected from virtual screening.

Safety ought to be great considered during the study. To ensure the safety of these 20 compounds, various types of toxicity indexes of the compounds and Tivantinib, such as developmental toxicity potential properties, rodent carcinogenicity (based on the U.S. National Toxicology Program dataset), as well as Ames mutagenicity were predicted using a computational method in the TOPKAT module (Table 3). Consequence indicated/illustrated 14 compounds were found to be nonmutagenic, and 5 compounds were found with no developmental toxicity potential. It is predicted that Tivantinib had higher rodent carcinogenicity in mice than in rats. In consideration of all the above results, ZINC000005879645 and ZINC000002528509 were determined to be the perfect lead compounds with non-CYP2D6 inhibitors, thus without hepatotoxicity, together with less Ames mutagenicity, developmental toxicity potential, and rodent carcinogenicity in comparison with other compounds. To sum up, ZINC000005879645 and ZINC000002528509 were regarded as safe drugs and chosen for the following study (Fig. 2).

**Table 3:** Predicted toxicities of compounds.

		Mouse NTP		Rat NTP			
Number	Compounds	Female	Male	Female	Male	Ames	DTP
1	ZINC000004654840	1	1	0	0.994	0.019	1
2	ZINC000004654839	1	1	0	0.994	0.019	1
3	ZINC000049784088	0.995	0	0	0.008	1	1
4	ZINC000001531664	0.999	1	0	1	0	1
5	ZINC000014811789	0.096	1	0.031	1	1	1
6	ZINC000004098302	0	1	1	1	0	0
7	ZINC000011616633	0	1	1	1	1	1
8	ZINC000014614772	0	1	1	1	0	0
9	ZINC000002528509	0.999	0.041	0	0.999	0.999	0.745
10	ZINC000028882432	0.057	1	0	0.987	0	1
11	ZINC000013328774	0.865	1	1	1	0.674	1
12	ZINC000002526388	0.999	0.041	0	0.999	0.999	0.745
13	ZINC000006073947	0.001	0.733	1	1	0	0
14	ZINC000002528510	0.999	0.036	0	0.999	0.999	0.769
15	ZINC000013130933	0	1	1	0.011	0	0
16	ZINC000004098631	0	0.117	1	1	0	1
17	ZINC000004654839	1	1	0	0.994	0.019	1
18	ZINC000031163978	1	0	1	0.719	0	0
19	ZINC000005879645	0.579	0.852	0.965	0.997	0.914	1
20	ZINC000001763468	1	1	0.998	1	0	1
21	Tivantinib	1	1	0	0	0	1

NTP, U.S. National Toxicology Program; DTP, developmental toxicity potential. NTP <0.3 (noncarcinogen); >0.8 (carcinogen). Ames <0.3 (nonmutagen); >0.8(mutagen). DTP <0.3 (nontoxic); >0.8 (toxic).

### 3.3 Analysis of ligand binding

**Figure 3:**
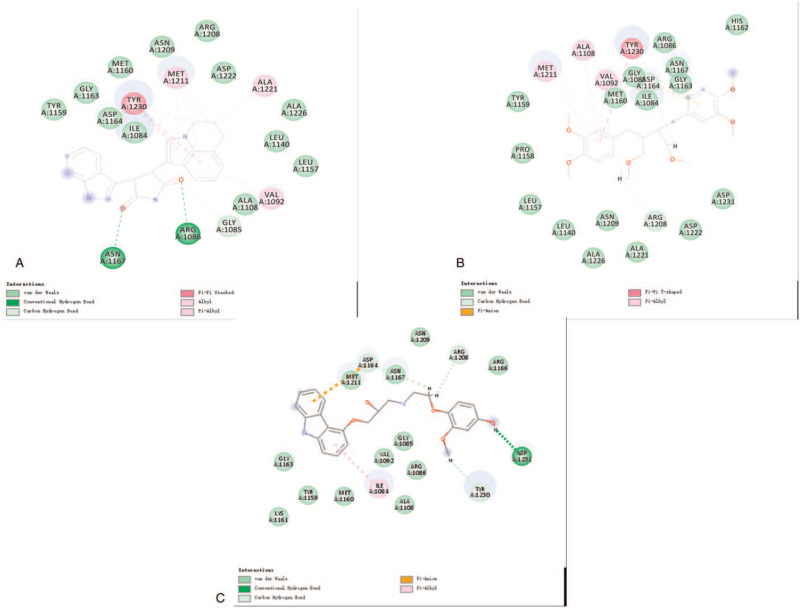
Schematic representation of intermolecular interaction of the predicted binding modes of (A) ZINC000005879645, (B) ZINC000002528509, and (C) Tivantinib.

**Figure 4:**
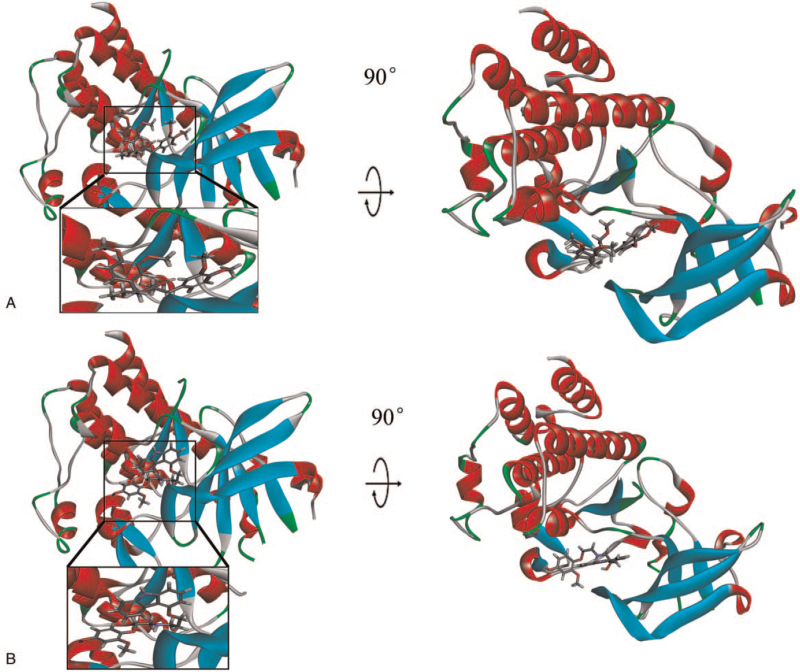
Schematic drawing of interaction between ligands and c-Met. The surface of binding areas was added. Blue represents positive charge and red represents negative charge; and ligands are shown in sticks, with the structure around the ligand-receptor junction shown in thinner sticks. (A) ZINC000005879645 and (B) ZINC000002528509.

To study ligand blinding mechanisms of these compounds with c-MET. ZINC000005879645 and ZINC000002528509 were docked into the molecule structure of c-MET by CDOCKER module, and CDOCKER potential energy was calculated and displayed as shown in Table 4. The CDOCKER potential energy of ZINC000008220033 and ZINC000001529323 were significantly lower than the reference ligand Tivantinib, which illustrated that c-MET may have a higher binding affinity with ZINC000008220033 and ZINC000001529323 than Tivantinib. Through a structural computation study, we also performed Hydrogen bonds interaction analysis (Figs. 3 and 4). Results illustrated that 9 pairs of hydrogen bonds of ZINC000005879645 with c-MET were formed. ZINC000002528509 formed 7 hydrogen bonds with c-MET. As for reference Tivantinib, it formed 13 hydrogen bonds with AKT1 (Table 5).

**Table 4:** Predicted CDOCKER potential energy of compounds with CMET.

Compound	-CDOCKER potential energy (kcal/mol)
ZINC000005879645	31.36
ZINC000002528509	26.7177
Tivantinib	19.944

**Table 5:** Hydrogen bond interaction parameters for each compound with CMET.

Receptor	Compound	Donor atom	Receptor atom	Distances (Å)
CMET	ZINC000005879645	A:ASP1164:OD1	ZINC000005879645:H34	2.39
		ZINC000005879645:O2	ZINC000005879645:H49	2.56
		A:ARG1208:O	ZINC000005879645:H53	2.56
		ZINC000005879645:O25	ZINC000005879645:H61	2.41
		ZINC000005879645	A:ASP1164:OD1	3.79
		ZINC000005879645	A:TYR1230	5.56
		A:VAL1092	ZINC000005879645	4.30
		A:ALA1108	ZINC000005879645	5.33
		A:MET1211	ZINC000005879645	4.94
	ZINC000002528509	A:ASP1231:OD2	ZINC000002528509:H36	2.05
		A:TYR1230:O	ZINC000002528509:H33	2.65
		A:ASP1164:OD1	ZINC000002528509:H39	2.60
		A:ARG1208:O	ZINC000002528509:H40	2.45
		ZINC000002528509	A:ASP1164:OD1	3.90
		ZINC000002528509	ZINC000002528509	5.66
		A:ILE1084	ZINC000002528509	4.45
	Tivantinib	Tivantinib:O5	A:ARG1086:HN	2.06
		Tivantinib:O1	A:ASN1167:HD21	2.90
		Tivantinib:O1	A:ASN1167:HD22	2.59
		Tivantinib:O5	A:GLY1085:HA1	2.75
		Tivantinib:O5	A:GLY1085:HA2	2.84
		Tivantinib	A:TYR1230	5.33
		A:TYR1230	Tivantinib	4.16
		Tivantinib	A:ALA1221	4.02
		A:MET1211	Tivantinib	4.32
		Tivantinib	A:TYR1230	4.39
		A:MET1211	Tivantinib	4.79
		A:VAL1092	Tivantinib	4.60
		A:MET1211	Tivantinib	4.69

### 3.4 Molecular dynamics simulation

**Figure 5:**
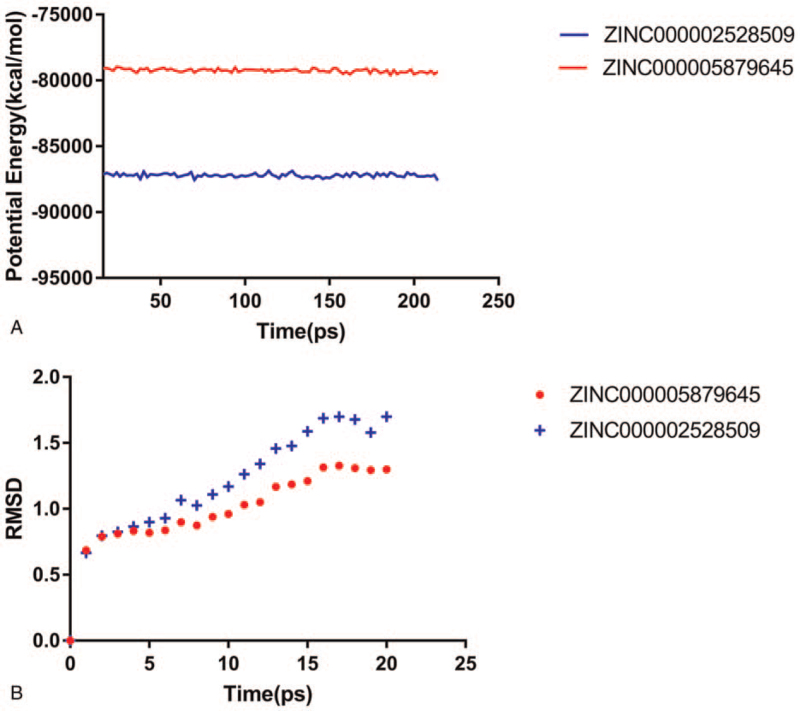
Results of molecular dynamics simulation of the compounds ZINC000005879645 and ZINC000002528509. (A) Average backbone root-mean-square deviation. (B) Potential energy. RMSD = root-mean-square deviation.

For the sake of estimating the stabilities of the ligand-c-MET complexes in the natural environmental circumstances, a molecular dynamics simulation module was established. The molecular docking experiment was used to get the original conformations through CDOCKER module. RMSD curves and potential energy chart of each complex were shown in Fig. 5. After 18 ps, the trajectories of each complex reached equilibrium. With time going by, the RMSD and potential energy of these complexes got stabilized gradually. Through molecular dynamics simulations, the hydrogen bond and p-dependent interactions between the compound and MGMT were validated that they contribute to the stability of these complexes. To sum up, ZINC000005879645 and ZINC000002528509 could interact with c-MET, and the complexes were stable in the natural environment which had an effect on c-MET.

### 3.5 U251 cells and c-MET expression in U251 cells

**Figure 6:**
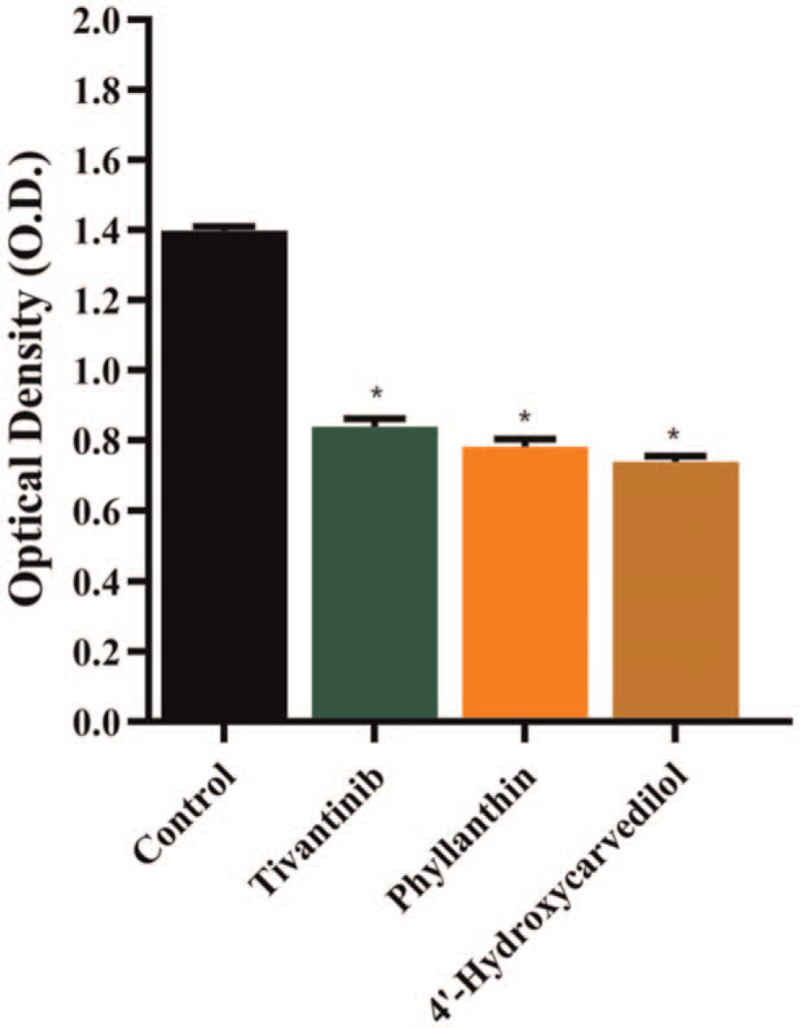
c-MET expression in U251 cells.

U251 cells were treated with Tivantinib, Phyllanthin (ZINC5879645), and 4′-Hydroxycarvedilol (ZINC2528509) for 72 hours. The level of c-MET secretion in, Tivantinib, Phyllanthin, and 4′-Hydroxycarvedilol group was found lower than that in the blank group, and the level of c-MET secretion in Phyllanthin and 4′-Hydroxycarvedilol group was lower than that in the Tivantinib group. It is suggested that Tivantinib, Phyllanthin, and 4′-Hydroxycarvedilol group has an inhibitory effect on the level of c-MET secretion of U251 cells compared with the blank group **(**Fig. 6).

## 4 Discussion

GBM is the primary brain tumor with the highest incidence in the skull, among which GBM has a very high degree of malignancy. Even after radiotherapy and chemotherapy, the median survival of patients is very short.^[21]^ c-MET, encoded by proto-oncogene. Met, is a highly binding receptor tyrosine kinase belonging to the RON subfamily and is the only known receptor of scatter factor or HGF. The interaction between c-MET and membrane receptors affects the role of signal molecules such as HGF/c-Met signal pathway and vascular endothelial growth factor and its receptor, and further affects the process of tumor invasion, metastasis, and neovascularization, which leads to the emergence of tumor drug resistance, and is an important reason for the failure of drug therapy.^[22]^ Therefore, the key to inhibiting tumor growth is to find an inhibitor of c-MET to limit its activity, so as to resist tumor growth. At present, there are more than a dozen small-molecule c-met kinase inhibitors in the preclinical or clinical research stage used as single-drug therapy or in combination with other targeted drugs or chemotherapy drugs to treat multiple malignant tumors. As a result, the results of this study can also be applied to other tumors.^[23]^

At the moment, crizotinib, a therapeutic drug, has been approved by the Food and Drug Administration as a dual inhibitor of c-MET-ALK. But it also has some limitations. First, Crizotinib is resistant and a crizotinib-resistant tumor became susceptible to crizotinib retreatment in a non-small cell lung cancer patient.^[24]^ In addition, crizotinib is cytotoxic. Although most of the adverse events such as digestive symptoms and visual impairment were grade 1, other serious adverse events such as interstitial pneumonia, liver injury, and QT prolongation were also reported.^[25]^ Tivantinib (ARQ-197) (selected as a reference drug in this study) is an oral, highly selective, non-ATP competitive c-MET receptor tyrosine kinase inhibitor.^[26]^ It has been shown to be effective as a second-line treatment for a variety of solid tumors in early clinical trials. Therefore, there is an urgent need to screen more compounds targeting c-MET for clinical applications.

Although phase I and II trials of Tivantinib in second-line use showed encouraging results, the manufacturer's press release said that the phase III study of Tivantinib in the treatment of hepatocellular carcinoma did not achieve the ultimate goal of improving overall survival.^[27]^ The future of Tivantinib, like many other drugs, depends on choosing the right patient. Some researchers have shown that patients with high expression of tumor MET showed great survival benefits compared with the placebo group;^[28]^ for patients with low expression of tumor MET, there was no difference between the Tivantinib group and placebo group, which indicated that Tivantinib still had a number of defects. Therefore, there is an urgent need to screen more compounds targeting c-MET for clinical applications.

In this study, LibDock, ADME/TOPKAT, CDOCKER, and Molecular Dynamics Simulation, 5 sections of Discovery Studio were used for virtual screening and analysis. As a result, 17931 biogenic-for-sale-named ligands were screened from the ZINC15 database for virtual screening. Compared with other compounds, compounds with a high LibDock score showed better energy optimization and a stable conformation. After the calculation of the module, 8827 compounds were found to be eligible to bind stably with c-MET than Tivantinib. The top 20 compounds were selected and pooled for further study on the basis of the LibDock score.

ADME and toxicity predictions of the selected compounds were used to evaluate the pharmacologic properties of these compounds. Outcomes illustrated that ZINC000005879645 and ZINC000002528509 were regarded as safe drugs and chosen for the following study. Since they could dissolve in water and also had a good absorption level. Additionally, they did not have hepatotoxicity and they were noninhibitors of CYP2D6. Besides, these 2 compounds were also found to have less mutagenicity, rodent carcinogenicity, and developmental toxicity potential compared with other compounds. Therefore, ZINC000005879645 and ZINC000002528509 were regarded as safe drug candidates. For another, the remaining drugs still had a possible function in drug development despite they possessed toxicities or negative effects. In view of all the results above, ZINC000005879645 and ZINC000002528509 were selected as ideal lead compounds and further analysis was performed.

The bonding mechanism and chemical bonds of the selected candidate compounds were also researched. CDOCKER module computation illustrated that the CDOCKER interaction energy of ZINC000008220033 and ZINC000001529323 was obviously lower than the reference ligand Tivantinib (−35 kcal/mol), which could indicate that these 2 compounds had a higher binding affinity with c-MET than Tivantinib.

Finally, their stabilities in the natural environment were investigated by molecular dynamics simulation. Calculation of RMSD and potential energy of these ligand-MGMT complexes demonstrated the trajectories of complexes reached equilibrium after 18 ps. With time going by, RMSD and potential energy of these complexes got stabilized gradually, which showed ZINC000005879645 and ZINC000002528509 could interact with c-MET and the complexes were stable in the natural environment. On account of the results, these 2 compounds could be used for drug development and refinement.

This study elucidated that the most important step in current drug designation was to screen ideal lead compounds. In this study, a battery of computer-aided virtual techniques was used to identify possible inhibitors of c-MET. LibDock is applied for structure-based screening followed by ADME and toxicity prediction.

Molecular docking was conducted to confirm the binding affinity mechanism between the ligand and c-MET. Molecular dynamics simulations were used to assess the stability of ligand-c-MET complexes. The results showed these 2 compounds might have the most potential effect on GBM. But it is all known that no single drug could be directly marketed without thousands of refinement and improvement. Therefore, their refinement and improvement are of great significance in the following research.

Then, ELISA in vitro was carried out to evaluate the effects of potential compounds in the study. c-MET is overexpressed in carcinomas and other solid tumors such as small cell lung cancer in human,^[29]^ ovarian tumors,^[30]^ esophageal cancer,^[31]^ and GBM.^[32]^ Therefore, efficient compounds inhibiting c-MET can be exploited therapeutically. We chose the expression level of c-MET as the evaluation indicator to access the drug effect. In ELISA assay, the level of c-MET expression in Phyllanthin (ZINC5879645) and 4′-Hydroxycarvedilol (ZINC2528509) group was lower than that in the Tivantinib group. Consequently, the results demonstrated that the effect of Phyllanthin and 4′-Hydroxycarvedilol was better than that of Tivantinib in anti-GBM.

Although this study was well-designed and precise measurements have been conducted, it still has some shortcomings. Further experiments, for instance, animal testing, need to be performed to confirm our results, and more indicators, such as half-maximal inhibitory concentration and half-maximal effective concentration, should be assessed in the future.

## 5 Conclusion

This study conducted a battery of computer-aided structural and chemistry techniques (eg, virtual screening, ADME, toxicity prediction, and molecule docking) to screen and identify the ideal lead compounds with functions to possibly inhibit c-MET. Two compounds, ZINC000005879645 and ZINC000002528509, were selected as safe drug candidates, and they played important role in c-MET inhibitor development. Besides, a list of drug candidates with pharmacologic properties was provided, which could make a great contribution to c-MET or other proteins in medication design and improvement.

## Author contributions

YY Hou and HQ Xie designed experiments; WZ Yang wrote the manuscript; JL Ge, JA Ren, JC Li, and Jing Wang carried out experiments; GJ Dou, ZY Zhang, and XH Wang analyzed experiments results.

**Writing – original draft:** Yuanyuan Hou, Haoqun Xie, Gaojing Dou.

**Writing – review & editing:** Yuanyuan Hou, Gaojing Dou, Wenzhuo Yang, Junliang Ge, Junan Ren, Juncheng Li, Jing Wang, Zhiyun Zhang, Xinhui Wang, Baolin Zhou.
